# Mitosis Phase Enrichment with Identification of Mitotic Centromere-Associated Kinesin As a Therapeutic Target in Castration-Resistant Prostate Cancer

**DOI:** 10.1371/journal.pone.0031259

**Published:** 2012-02-17

**Authors:** Kanishka Sircar, Heng Huang, Limei Hu, Yuexin Liu, Jasreman Dhillon, David Cogdell, Armen Aprikian, Eleni Efstathiou, Nora Navone, Patricia Troncoso, Wei Zhang

**Affiliations:** 1 Department of Pathology, The University of Texas MD Anderson Cancer Center, Houston, Texas, United States of America; 2 Department of Computer Science and Engineering, The University of Texas Arlington, Arlington, Texas, United States of America; 3 Department of Genitourinary Medical Oncology and the David H. Koch Center for Applied Research of Genitourinary Cancers, The University of Texas MD Anderson Cancer Center, Houston, Texas, United States of America; 4 Division of Urology, McGill University, Montreal, Quebec, Canada; Florida International University, United States of America

## Abstract

The recently described transcriptomic switch to a mitosis program in castration-resistant prostate cancer (CRPC) suggests that mitotic proteins may be rationally targeted at this lethal stage of the disease. In this study, we showed upregulation of the mitosis-phase at the protein level in our cohort of 51 clinical CRPC cases and found centrosomal aberrations to also occur preferentially in CRPC compared with untreated, high Gleason–grade hormone-sensitive prostate cancer (*P*<0.0001). Expression profiling of chemotherapy-resistant CRPC samples (n = 25) was performed, and the results were compared with data from primary chemotherapy-naïve CRPC (n = 10) and hormone-sensitive prostate cancer cases (n = 108). Our results showed enrichment of mitosis-phase genes and pathways, with progression to both castration-resistant and chemotherapy-resistant disease. The mitotic centromere-associated kinesin (MCAK) was identified as a novel mitosis-phase target in prostate cancer that was overexpressed in multiple CRPC gene-expression datasets. We found concordant gene expression of MCAK between our parent and murine CRPC xenograft pairs and increased MCAK protein expression with clinical progression of prostate cancer to a castration-resistant disease stage. Knockdown of MCAK arrested the growth of prostate cancer cells suggesting its utility as a potential therapeutic target.

## Introduction

Prostate cancer is the second leading cause of cancer-specific mortality in the United States [1], with most deaths occurring after failure of androgen-deprivation therapy when the tumor grows as castration-resistant prostate cancer (CRPC), killing the patient within a median period of 18 months. Docetaxel-based chemotherapy has been established as the standard of care in CRPC, with a small but definite survival benefit [2], [3]. Identifying targets for this terminal castration-resistant and chemotherapy-resistant phase of disease thus represents an unmet need in prostate cancer [4], [5].

An important recent advance in this field was the demonstration that the androgen receptor activates a distinct mitosis-phase transcriptional program in CRPC but not in hormone-sensitive prostate cancer (HSPC) [6], partially explaining the relative success of docetaxel, an anti-mitotic chemotherapeutic agent used to treat CRPC. Those results also suggest that other mitosis-phase proteins may be potential targets for therapies treating this stage of disease.

We sought to first validate the upregulation of the mitosis-phase transcriptional program at the protein level in CRPC using clinical CRPC and high-grade HSPC cases. We next compared the transcriptomic profiles of localized CRPC samples that were chemotherapy-resistant with CRPC chemotherapy-naïve tumors and hormone-sensitive prostate cancers. Pathway analysis of data from our in-house specimens and publicly available databases identified enrichment of mitosis-related genes as the disease progressed to both a castration-resistant and chemotherapy-resistant stage. The mitotic centromere-associated kinesin (MCAK), whose gene expression was upregulated in multiple CRPC chemotherapy-resistant datasets, was selected for clinical and functional validation. MCAK protein expression was associated with clinical progression of prostate cancer, and its knockdown arrested the growth of prostate cancer cells.

## Results

### CRPC tumors show augmented mitosis-phase protein expression and centrosomal aberrations compared with high-grade HSPC

**Figure 1:**
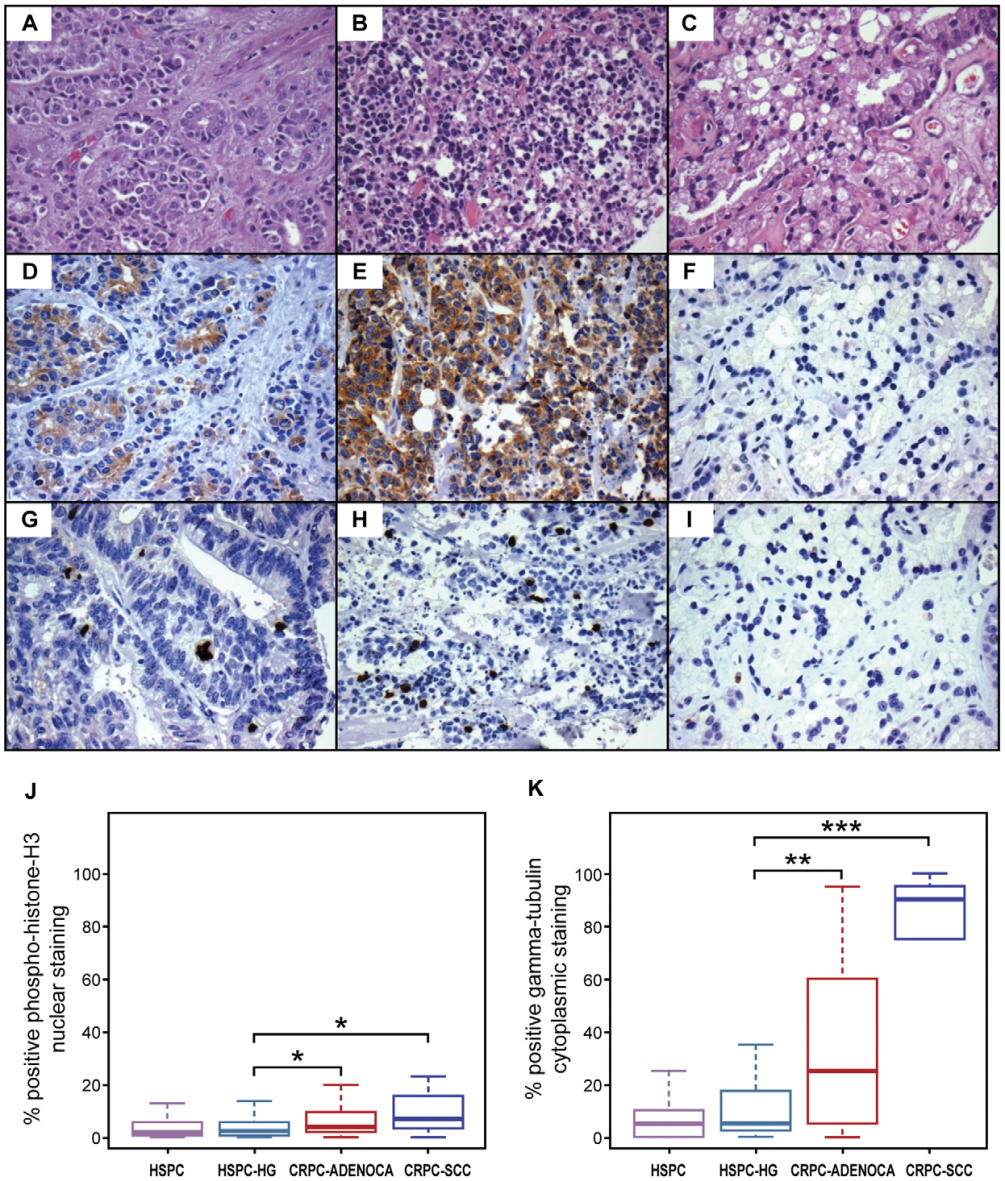
Increased mitosis-phase and centrosomal protein expression in castration-resistant prostate cancer compared with high-grade hormone-sensitive prostate cancer. Hematoxylin and eosin (H&E)-stained sections showing castration-resistant adenocarcinoma (A), castration-resistant small cell carcinoma (B), and hormone-sensitive high-grade prostate carcinoma (C). Castration-resistant adenocarcinoma immunostained with gamma tubulin (D) and p-histone H3 (G). Castration-resistant small cell carcinoma immunostained with gamma tubulin (E) and p-histone H3 (H). Hormone-sensitive high-grade prostate carcinoma immunostained with gamma tubulin (F) and p-histone H3 (I). Asterisks indicate significantly increased labeling for the mitosis-phase marker p-histone H3 (J) and centrosomal marker gamma-tubulin (K) in castration-resistant prostate carcinoma compared to hormone-sensitive high-grade prostate carcinoma.

To validate the putative transcriptomic switch to a mitosis-phase program in CRPC at the protein level, we used nuclear phosphohistone H3 immunostaining as a mitosis-phase marker, as histones are phosphorylated at the end of prophase and their phosphorylation peaks at metaphase [7]. We quantitatively examined tissue-microarray sections of HSPC and CRPC with the Ariol image analysis system. Our tissue arrays included 557 cores and >250,000 nuclei from 75 clinical cases. Our results showed significant upregulation of phosphohistone H3 in castration-resistant prostatic carcinoma compared with HSPC (Figures 1G–1I and Table S2). These results provide evidence that mitosis-phase proteins are upregulated in CRPC. Importantly, high-grade hormone-sensitive tumors with Gleason grades of 4/5 also showed significantly lower phosphohistone H3 expression than castration-resistant tumors of the adenocarcinoma or small cell carcinoma histologies (P<0.0001) (Figures 1G–1J and Table S2). Given the vastly different transcriptional profiles and far more aggressive clinical behavior of high Gleason–grade (Gleason grades 4/5) prostate cancer compared with low Gleason–grade (Gleason grade 3) prostate cancer, our data underscores the distinctiveness of CRPC from all histological subtypes of HSPC.

In studying the mitosis phase, we also examined centrosome abundance, since centrosomes are key players in cell division. Gamma tubulin, a microtubule nucleator and marker of centrosome amplification, showed significantly increased cytoplasmic labeling in castration-resistant disease when compared with HSPC (P<0.0001) and high-grade HSPC (P = 0.008) (Figures 1D–1F and 1K and Table S3). This finding is in keeping with the known role of centrosome amplification and genetic instability in the progression of other cancers [8], [9], [10], [11].

### Upregulation of the mitotic apparatus in CRPC tumors with progression to castration resistance and chemotherapy resistance

We macrodissected tumor cells that were castration-resistant and chemotherapy-resistant from localized non-metastatic frozen surgical samples from 20 unique patients and five xenografts derived thereof (Table S1). Transcriptomic profiling of total RNA was performed on those tumor samples and five benign prostate samples that were used to normalize our expression data. Our array raw data is submitted to GEO (GSE 33277). As our data were derived from both terminal castration-resistant and docetaxel chemotherapy–resistant tumors, we compared the mRNA expression profile of these samples to that of samples from earlier stages of prostate cancer progression. Specifically, we compared our data to public gene-expression data derived from untreated HSPC (GSE 21032, n = 108) and chemotherapy-naïve CRPC (GSE 6811, n = 10). Such meta-analyses are challenging given the numerous variables that can affect the intensity of the hybridization signal. For a more reliable comparison, we specifically chose datasets of localized, non-metastatic HSPC and chemotherapy–naïve CRPC samples that had been normalized to data from pooled benign prostate samples or where gene-expression data from benign prostate samples that permitted such normalization was provided. As expected, we found overexpression of mitosis-phase genes and pathways relative to untreated HSPC samples from the Memorial Sloan Kettering Cancer Center dataset (GSE 21032). These results are in agreement with previous reports [6] but use independent datasets and methodologies.

**Figure 2:**
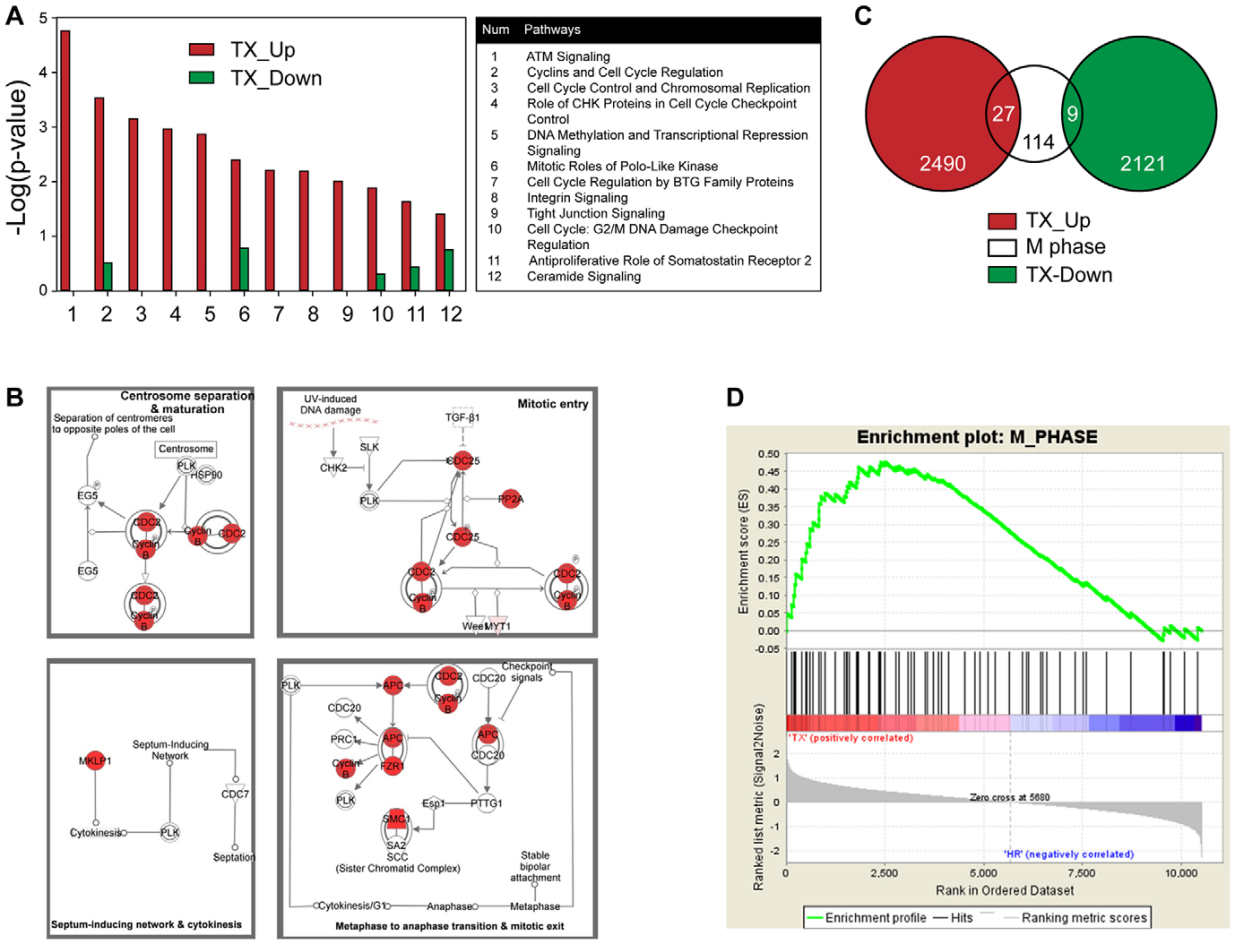
Enhancement of mitosis-phase genes and pathways with progression to chemotherapy-resistant disease. (A) Cell-cycle regulation pathways are significantly upregulated in the CRPC chemotherapy-resistant (TX) group compared with the CRPC chemotherapy-naïve group. (B) Ingenuity Pathway Analysis.(IPA) of the upregulated gene set demonstrates that the canonical pathway of mitotic roles of polo-like kinase is significantly associated with the CRPC chemotherapy-resistant group (*P* = 0.004). The *P* value is calculated by Fisher's Exact Test. The genes that are upregulated in the CRPC chemotherapy-resistant group and are involved in this pathway are color-coded in red. The other genes that are included in this pathway but are not in the upregulated gene set are indicated in white. (see text for identification of upreguated gene set in details). (C) Significant overlap of the upregulated gene set in CRPC chemotherapy-resistant tumors with M-phase (n = 27, *P* = 5.00E-7) compared with the downregulated gene set (n = 9, *P* = 0.416). (D) GSEA indicated that M-Phase was significantly enriched in the CRPC chemotherapy-resistant group at FDR<10%.

The University of Tokyo dataset (GSE 6811) is one of very few that includes CRPC chemotherapy-naïve samples. Significance of microarray (SAM) analysis comparing the CRPC chemotherapy-naïve group with our CRPC chemotherapy-resistant group identified two informative gene sets that consisted of 2,490 upregulated and 2,121 downregulated genes in the chemotherapy-resistant group. Pathway analysis of these two gene sets performed using Ingenuity Pathway Analysis demonstrated that several cell-cycle regulation pathways were significantly enriched in the upregulated gene set (Figure 2A). In particular, the chemotherapy-resistant group exhibited significant overexpression of the mitosis-involved pathway (Figure 2B), as compared with the chemotherapy-naïve group (Figure S1). Furthermore, overlap analysis of these two gene sets, as assessed by a hypergeometric distribution model (Figure 2C) using mitosis-phase (M-phase) genes obtained from the MSigDB database (http://www.broadinstitute.org/gsea/msigdb/index.jsp), showed significant overlap with only those genes that were upregulated in the chemotherapy-resistant group (n = 27; *P* = 5.0×10^−7^). The genes downregulated in the chemotherapy-resistant group did not significantly overlap with the M-phase genes (n = 9; *P* = 0.416). This observation was supported by gene set–enrichment analysis (GSEA; http://www.broadinstitute.org/gsea/index.jsp, Version 3.7), which showed that the M-phase gene set was significantly enriched in the chemotherapy-resistant group at a false discovery rate (FDR) of <10% (Figure 2D). These analyses suggest that mitosis-associated genes and pathways may be involved in mediating chemotherapy resistance.

### Mitotic centromere-associated kinesin is associated with progression to therapy resistance, while its silencing inhibits prostate cancer cell growth

**Figure 3:**
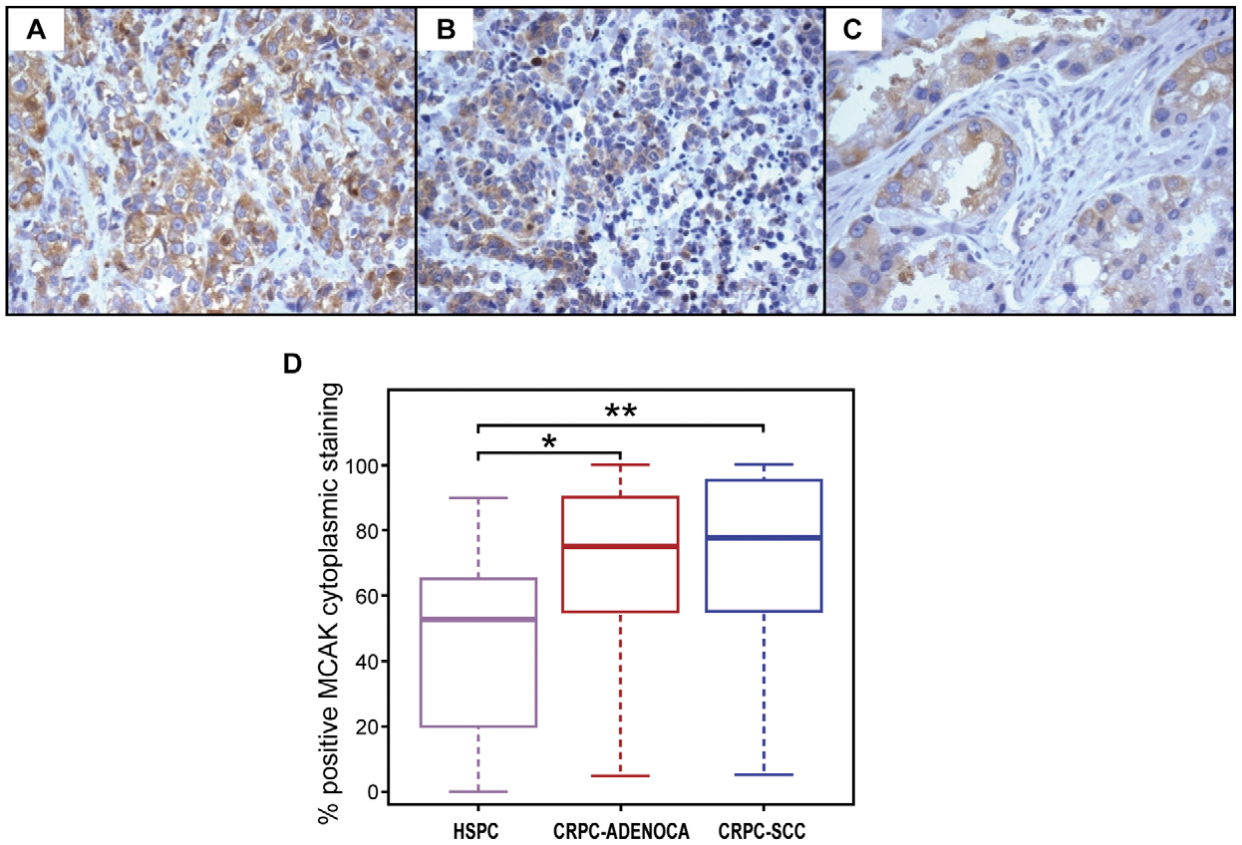
Overexpression of MCAK with clinical progression of prostate cancer. **MCAK**-immunostained sections of castration-resistant adenocarcinoma (A), castration-resistant small cell carcinoma (B), and hormone-sensitive prostate carcinoma (C). D) Significantly increased labeling for the mitotic centromere-associated protein MCAK with progression to CRPC, indicated by asterisks.

Examination of the specifically altered mitotic regulators in our transcriptomic data led us to identify genes coding for the mitotic kinesin family of motor proteins as being upregulated in CRPC chemotherapy-resistant samples. These mitotic kinesins were also upregulated in two other large CRPC chemotherapy-resistant datasets from the University of Michigan (GDS 1439) and Memorial Sloan Kettering Cancer Center (GSE 21032). We selected the mitotic centromere-associated kinesin (MCAK) as a target in prostate cancer because it was novel and there was marked overexpression of the MCAK gene (*P* = 1.55×10^−15^) in localized CRPC from the MD Anderson Cancer Center dataset as compared to hormone sensitive prostate cancer cases (n = 108) from the Memorial Sloan Kettering Cancer Center dataset (GSE 21032). MCAK was also upregulated with progression to metastatic CRPC within the Memorial Sloan-Kettering Cancer Center cohort (*P* = 3.73×10^−10^) (Figure S2). Moreover, we found concordant expression of MCAK in both our human parent CRPC tumors and murine xenografts derived from those tumors (*r* = 0.428) as shown in Figure S3. We subsequently measured MCAK protein expression by immunohistochemical analysis of tissue microarrays of independent hormone-sensitive (n = 38) and castration-resistant (n = 51) cases. We found MCAK cytoplasmic staining to be significantly associated with clinical progression to castration-resistant disease (P<0.0001) (Figures 3A–D and Table S4).

**Figure 4:**
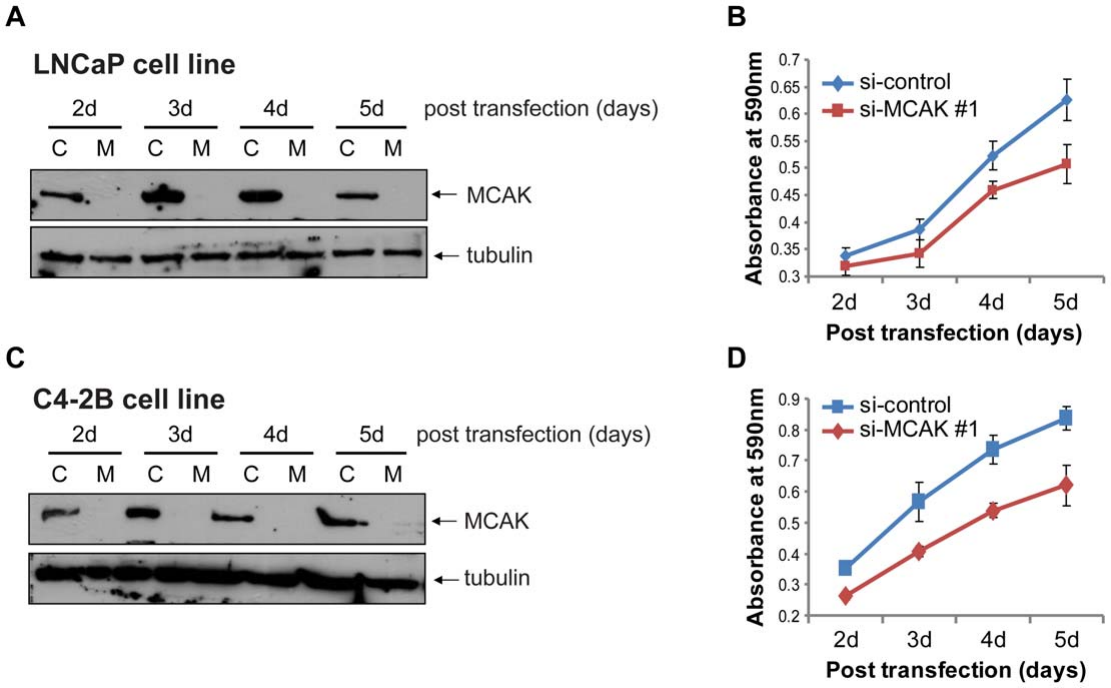
Growth inhibition of LNCaP and C4-2B cells by si-MCAK. A) Western blot confirming knockdown of MCAK by si-MCAK#1. Whole-cell lysates from LNCaP cells transfected with si-control (C) or si-MCAK #1 (M) were collected at different time points after transfection, as indicated. Tubulin was used as the loading control. B) MTT cell growth assay. LNCaP cells were treated the same as in A), and after a 24-h transfection, 4,000 cells were seeded into each well of a 96-well plate (n = 10 for each group) and the MTT assay was performed in a 24 h interval. C) Western blot confirming knockdown of MCAK with si-MCAK#1. Whole-cell lysates from C4-2B cells transfected with si-control (C) or si-MCAK #1 (M) were collected at different time points after transfection, as indicated. Tubulin was used as the loading control. D) MTT cell growth assay. C4-2B cells were transfected with si-control and si-MCAK #1, respectively. After a 24-h transfection, 4,000 cells were seeded into each well of a 96-well plate and incubated for different time periods, as indicated, followed by the MTT assay (n = 10 for each group).

To determine the effects of knocking down MCAK in HSPC and CRPC, we used two distinct sets of small interfering RNA (siRNA) specific for MCAK to silence its expression in hormone-sensitive LNCaP and castration-resistant chemotherapy-naïve C4-2B prostate cancer cell lines. Both CRPC and HSPC cell lines showed abundant intrinsic MCAK protein expression and both showed growth inhibition after 3 days of MCAK knockdown with the first siRNA(si-MCAK #1) as assessed by an MTT assay (Figure 4A–D) (C4-2B: 3 d, P = 5.45×10^−7^; 4 d, P = 4.85×10^−10^; 5 d, P = 3.78×10^−8^; LNCaP: 3 d, P = 1.43×10^−3^; 4 d, P = 3.86×10^−5^; 5 d, P = 1.48×10^−5^). Similar results from the second set of siRNA (si-MCAK #2) are shown in Figure S4.

## Discussion

In this study, we have extended the findings of Wang et al [6] by validating, at the protein level, the transcriptomic switch to a mitosis program in an independent set of CRPC clinical samples and showing that differences between castration resistant and untreated hormone sensitive prostate cancers are maintained even when comparing high Gleason grade prostate cancers. This is an important finding given the different transcriptomic programs seen in prostate cancers with low and high Gleason grades [12] that reflect their dramatically differing clinical behavior. We have also shown centrosomal aberrations to occur preferentially in CRPC, in keeping with the abnormal cell division seen in other advanced cancers. These results support the concept that CRPC is biologically distinct and not merely an extension of high-grade hormone sensitive prostate cancer, despite their histological similarities and propensity for clinical aggressiveness.

By comparing transcriptomic profiles of CRPC surgical samples that were chemotherapy-naïve with our CRPC chemotherapy-resistant data, we found enrichment of mitotic-phase proteins and pathways in the chemotherapy-resistant tumors. Among the mitosis-related genes, we selected the mitotic kinesin family of motor proteins for further study—in particular MCAK, which has not been previously studied in prostate cancer. Mitotic kinesins are known to play a crucial role in regulating the mitotic spindle apparatus during cell division. They are involved in cellular proliferation and are regulated by aurora-B kinase [13]. Progression of lung, brain, and colorectal cancer [14] [15] has been associated with augmented kinesin expression, while its inhibition has resulted in cell-cycle arrest in the mitosis phase [16], [17]. Mitotic centromere-associated kinesin is plentiful in centrosomes, depolymerizes microtubules, and mediates the transition from prometaphase to metaphase [18], among other essential functions related to proper chromosome segregation [19] during cell division. Overexpression of mitotic kinesins, which are postulated to work by countering the microtubule-stabilizing effect of taxane-based chemotherapy, has been linked to docetaxel resistance in breast cancer [20], and MCAK overexpression has been causally related to paclitaxel resistance [21].

Targeting mitotic kinesins is thus emerging as an attractive treatment modality in cancers that are refractory to taxane-based chemotherapies that act upon the mitotic spindle, since inhibition of kinesin motor proteins does not directly target microtubules. Moreover, mitotic kinesins are not expressed by neurons [22] and are poor substrates for the P-glycoprotein efflux pump [23], which result in docetaxel's dose-limiting neurotoxicity and multidrug resistance. Hence, mitotic kinesin inhibition is hypothesized to induce mitosis-specific cell-cycle arrest that may complement current chemotherapeutic protocols with fewer side effects [18].

Different mitotic kinesins serve specific functions, and the role of these proteins in prostate cancer has only been explored with respect to the most well-established member of this family, kinesin spindle protein (KIF11/Eg5), whose silencing has been shown to inhibit the growth of LNCaP and PC3 cell lines *in vitro* and *in vivo* [24], [25]. Early-phase clinical trials in CRPC patients using the first generation Eg5 inhibitor, ispinesib, have met with limited success [26] [27]. However, newer-generation Eg5 inhibitors have been reported to exhibit greater efficacy *in vitro* [28], and a third clinical trial is currently recruiting patients from a range of malignancies, including castration resistant prostate cancer (#NCT01065025).

In summary, we have shown enrichment of mitosis-phase pathways and proteins as prostate cancer progresses to both a castration-resistant and chemotherapy-resistant state. We examined the role of MCAK for the first time in prostate cancer and showed that MCAK expression correlates with clinical progression of this disease. Moreover, we demonstrated growth inhibition in MCAK-silenced castration-resistant prostate cell lines. Together, our results suggest that MCAK is a functionally important protein in terminal prostate cancer. Follow-up studies based on the present report should include using synthetic inhibitors directed against MCAK (e.g., patent #7294640, Merck) in preclinical models and in future clinical trials of prostate cancer.

## Materials and Methods

### Ethics statement

Hormone-sensitive and castration-resistant prostate cancer tissue samples were obtained with approval from the from the institutional review boards of The University of Texas MD Anderson Cancer Center (Houston, Texas) and McGill University (Montreal, QC, Canada). The tissue specimens used to develop MDA PCa 79, MDA PCa 117-9, MDA PCa 124, MDA PCa 130, MDA PCa 149-1, and MDA PCa 180-30 xenografts were derived from tumor samples of surgical specimens (cystoprostatectomy, radical prostatectomy, or pelvic exenteration) of a progressive primary castration-resistant prostate cancer. Written informed consent had been obtained from patients before sample acquisition, and samples were processed according to a protocol approved by MD Anderson institutional review board, including approval from the animal care and use committee. The lab protocol numbers that covered the use of these samples are MDACC LAB 03-0336 and LAB 09-0213.

### Patient tissue samples

Molecularly profiled tissues were derived from MD Anderson frozen tumor samples from patients with castration-and chemotherapy-resistant prostate cancer (n = 20) and from benign prostatic tissue controls (n = 5). The castration-resistant chemotherapy-resistant samples were taken from salvage cystoprostatectomies or pelvic exenterations. Murine xenografts derived from five of these 20 tumors showing adenocarcinoma histology were also assessed. Clinical data of the genomically profiled samples are included in Table S1. Four tissue microarrays were used to validate genomic data and consisted of formalin-fixed paraffin-embedded samples from 58 patients with HSPC and 51 patients with CRPC.

### Expression profiling and analysis

Tumor mRNA expression was assessed using a human whole-genome oligonucleotide microarray kit from Agilent (prod #G4112F) according to the manufacturer's protocol and as described previously [29]. Our CRPC data was normalized to the mean gene-expression values from five benign prostate samples to find upregulated or downregulated genes using a log ratio value >0.3 as the cut-off for informative genes present in more than two-thirds of cases. We compared our expression profiling data to the University of Michigan (GDS 1439), University of Tokyo (GSE 6811), and Memorial Sloan Kettering Cancer Center (GSE 21032) datasets. For these datasets, normalization was performed against their internal controls (benign samples).

### Immunohistochemical analysis

Standard 3,3′-diaminobenzidine (DAB) immunohistochemical analyses of tissue microarrays were performed with a Dako AutoStainer Plus (Dako) using a mouse anti-human monoclonal MCAK antibody (Abgent AT2621a) at 1∶50 dilution, a phospho-histoneH3 antibody (Santa Cruz SC-8656) at 1∶100 dilution, and a gamma-tubulin antibody (Abcam AB27074) at 1∶400 dilution, as described previously [30]. Staining for gamma-tubulin and MCAK was evaluated manually by assigning a percent positive value to each case based on the aggregate immunohistochemical labeling of all tissue cores belonging to that case.

### Automated image analysis

The phosphohistone H3–positive nuclei were quantitatively assessed using automated image analysis with Ariol software from Applied Imaging (Genetix Ltd.). A custom-made, automated nuclear quantitation algorithm using the Ariol software was prepared based on color variations of the brown stain in the DAB-positive cells versus the negative nuclear hematoxylin background to achieve a percent positive per pre-selected area of representation on each slide.

### Cell culture

C4-2B cells deposited at MD Anderson were developed after serial passage of LNCaP cells in castrated hosts when the cells were no longer responsive to androgen manipulation in animals (PMID: 8168083). LNCaP cells were obtained from American Type Culture Collection (Manassas, VA, USA). Both cell lines were cultured in RPMI1640 medium supplemented with 10% fetal bovine serum at 37°C with an atmosphere of 5% CO_2_.

### Xenograft studies

The tissue specimens used to develop the MDA PCa 79, MDA PCa 117-9, MDA PCa 124, MDA PCa 130, and MDA PCa 149-1 xenografts were residual tissues from surgical resection (cystoprostatectomy or pelvic exenteration) of progressive primary CRPC. Small tumor pieces were implanted into subcutaneous pockets of 6- to 8-week-old male CB17 SCID mice (Charles River Laboratories). Tumors developed within 6 months and were maintained in the mice, as the cells could not be grown *in vitro*. The passage and tumor processing methods used were the same as those reported previously (PMID: 18618013).

### SiRNA transfection

The LNCaP or C4-2B cells were seeded into a 6-well plate at a density of 5×10^4^ cells/well and cultured in RPMI1640 medium supplemented with 10% FBS under normal tissue culture conditions. After 20–24 h incubation, siRNA transfection was performed using LipofectamineRNAiMax Reagent from Invitrogen (Invitrogen, cat # 56532) by following the manufacturer's protocol. Two sets of si-MCAK siRNA were used for the experiments. si-MCAK #1 was purchased from Ambion (Ambion, cat # 4390824) and the si-MCAK #2 was purchased from Dharmacon (Chicago, IL, USA, cat # L-004955-00). The Universal Negative control #1 siRNA from Sigma (cat # SIC-001) was used as a control. The final concentration of the siRNA was 50 nM.

### Western blot analysis

MCAK monoclonal antibody was obtained from Abgent (cat # AT2621a) and both β-actin (cat # sc-1616) and β-tubulin (cat # sc-9104) were purchased from Santa Cruz Biotechnology (Santa Cruz, CA, USA). Western blotting was performed as described [31]. Briefly, cell extracts containing 30 µg of protein were resolved by 10% SDS-PAGE electrophoresis, transferred to Hybond ECL nitrocellulose membranes (Amersham Pharmacia Biotech) or polyvinylidene fluoride membranes (Cat # IPVH20200) from Millipore (Millipore Corporation, cat. # IPVH20200), blocked in 5% nonfat milk in 1× Tris-buffered saline plus 0.05% tween-20 (pH 8.0) and probed with primary antibodies at concentrations of 1∶1000 for MCAK and 1∶2500 for β-actin or β-tubulin. The secondary antibodies were used at concentrations of 1∶10,000. The proteins were visualized using the SuperSignal West Pico Chemiluminescent Substrate (Prod # 34708) or SuperSignal West Femto Maximum Sensitivity Substrate (Prod # 34096) from Pierce (Pierce Chemical, Rockford, IL, USA).

### Cell proliferation assays

Cell proliferation was determined by an MTT absorbance assay. The cells transfected with either control siRNA (si-control) or MCAK siRNA (si-MCAK) were trypsinized from the dishes at 24 h post transfection and 4,000 cells in 200 µl complete medium were seeded into each well of a 96-well plate (n = 10). The cells were allowed to attach for 24 h in the complete medium, and the MTT assay was performed at 24 h intervals subsequently. After a 2.5 h incubation with 50 uM MTT reagent in normal cell culture conditions, the wells were emptied by vacuum aspiration, and 200 µl DMSO was added to each well. The absorbance was measured at 590 nm with a microplate reader (MRX; Danatech Laboratory).

### Statistical analysis

Data are expressed as the mean +/− s.d. Statistical analyses were performed using Student's *t*-test and the Wilcoxon rank-sum test. Differences in means were evaluated using a two-tailed *t*-test, assuming unequal variances. A P value≤0.05 was considered statistically significant.

## Supporting Information

Figure S1 **Mitosis pathways are not associated with the downregulated gene set in CRPC chemotherapy-resistant disease.** Ingenuity Pathway Analysis (IPA) of the downregulated gene set shows that the canonical pathway of mitotic roles of polo-like kinase is not significantly associated with the CRPC chemotherapy-resistant group (*P* = 0.16). The *P* value is calculated by Fisher's Exact Test. The genes that are downregulated in the CRPC chemotherapy-resistant group and are involved in this pathway are color-coded in green. The other genes that are included in this pathway but are not included in the downregulated gene set are indicated in white.
(TIF)

Figure S2 **Increased MCAK transcript levels with progression to CRPC.** (A) MCAK shows transcriptomic upregulation in localized CRPC (*P* = 1.55×10^−15^) and (B) metastatic CRPC (*P* = 3.73×10^−10^) compared to HSPC.
(TIF)

Figure S3 **Concordance of MCAK gene expression between CRPC mouse xenografts (MDA-79, MDA-117, MDA-130, MDA-149) and their corresponding human parent tumors (** ***r*** **= 0.428).**
(TIF)

Figure S4 **Growth inhibition of LNCaP and C4-2B cells by si-MCAK #2.** A) Western blot confirming knockdown of MCAK by si-MCAK #2. Whole-cell lysates from LNCAP cells transfected with si-control (C) or si-MCAK #2 (M) were collected at different time points after transfection, as indicated. Actin was used as the loading control. B) MTT cell growth assays. LNCaP cells were treated the same as in A), and after a 24 h transfection, 4,000 cells were seeded into each well of a 96-well plate (n = 6 for each group) and followed by MTT assay. C) Western blot confirming knockdown of MCAK with si-MCAK #2. Whole-cell lysates from C4-2B cells transfected with si-control (C) or si-MCAK #2 (M) were collected at different time points after transfection, as indicated. Actin was used as the loading control. * indicates non-specific bands. D) MTT cell growth assays. C4-2B cells were transfected with si-control and si-MCAK #2, respectively. After a 24 h transfection, 4,000 cells were seeded into each well of a 96-well plate and incubated for different time periods, as indicated, followed by the MTT assay (n = 5 for each group).
(TIF)

Table S1 **Abbreviations: CXPX – cystoprostatectomy; PE – pelvic exenteration; TURP – transurethral resection; TAB – total androgen blockade (lupron + casodex); LHRH – lupron; ORCH – bilateral orchiectomy; XRT – radiation therapy.** * Small cell carcinoma component was profiled from a mixed histology small cell/adenocarcinoma tumor.
(DOC)

Table S2 **Abbreviations: HSPC – hormone sensitive prostate cancer; HSPC-HG – hormone sensitive prostate cancer of high histologic grade (Gleason patterns 4/5); CRPC-adeno – castration resistant prostate cancer with adenocarcinoma histology; CRPC-SCC – castration resistant prostate cancer with small cell carcinoma histology.**
(DOC)

Table S3 **Abbreviations: HSPC – hormone sensitive prostate cancer; HSPC-HG – hormone sensitive prostate cancer of high histologic grade (Gleason patterns 4/5); CRPC-adeno – castration resistant prostate cancer with adenocarcinoma histology; CRPC-SCC – castration resistant prostate cancer with small cell carcinoma histology.**
(DOC)

Table S4 **Abbreviations: HSPC – hormone sensitive prostate cancer; CRPC-adeno – castration resistant prostate cancer with adenocarcinoma histology; CRPC-SCC – castration resistant prostate cancer with small cell carcinoma histology.**
(DOC)
