# Supplementary figures and images for: Mitosis Phase Enrichment with Identification of Mitotic Centromere-Associated Kinesin As a Therapeutic Target in Castration-Resistant Prostate Cancer
Source: PLoS One. 2012 Feb 17;7(2):e31259. doi: 10.1371/journal.pone.0031259 (PMC3281954; doi:10.1371/journal.pone.0031259)

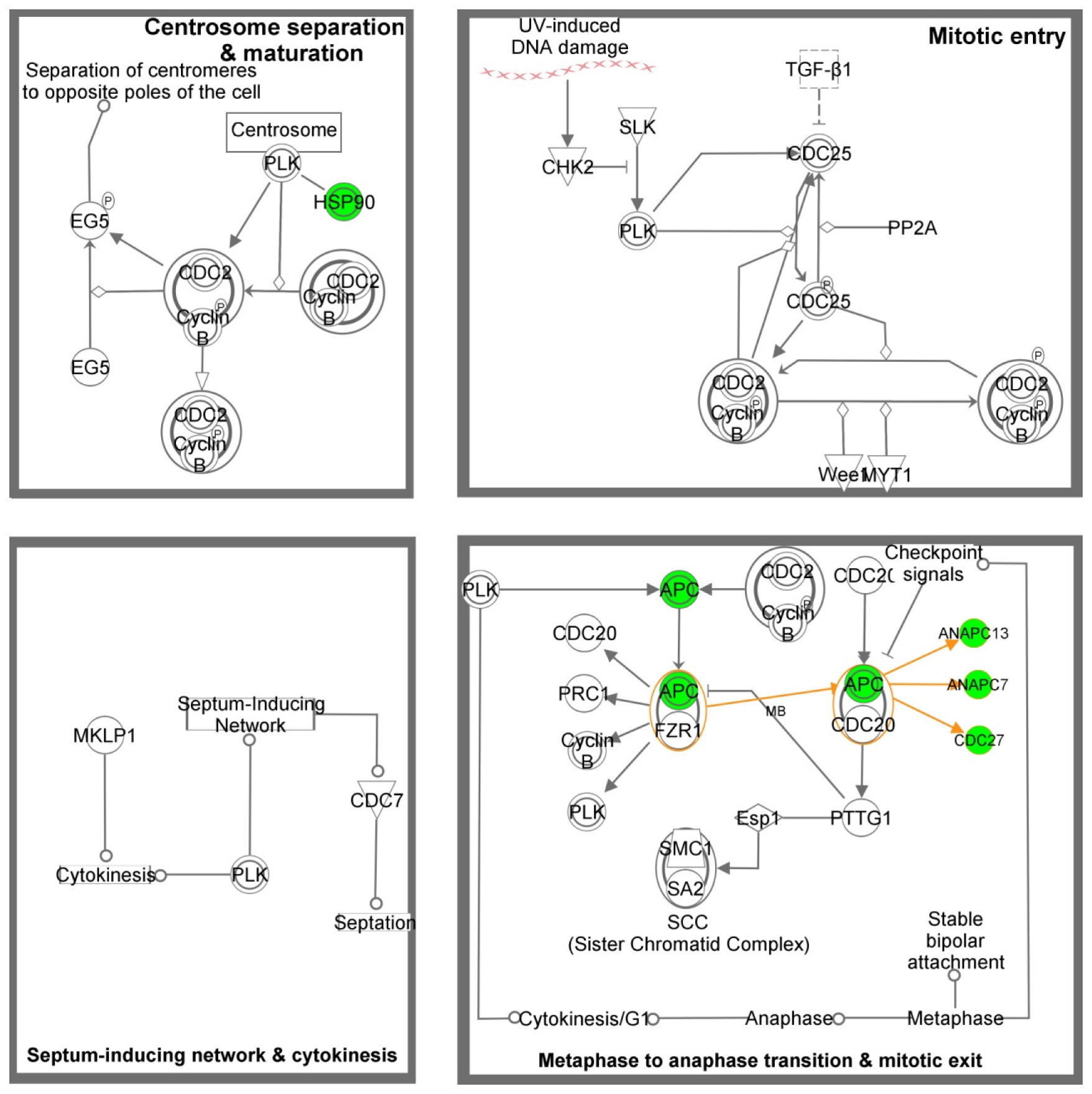

Supplement: Figure S1 — Mitosis pathways are not associated with the downregulated gene set in CRPC chemotherapy-resistant disease. Ingenuity Pathway Analysis (IPA) of the downregulated gene set shows that the canonical pathway of mitotic roles of polo-like kinase is not significantly associated with the CRPC chemotherapy-resistant group (P = 0.16). The P value is calculated by Fisher's Exact Test. The genes that are downregulated in the CRPC chemotherapy-resistant group and are involved in this pathway are color-coded in green. The other genes that are included in this pathway but are not included in the downregulated gene set are indicated in white. (TIF) [file pone.0031259.s001.tif]

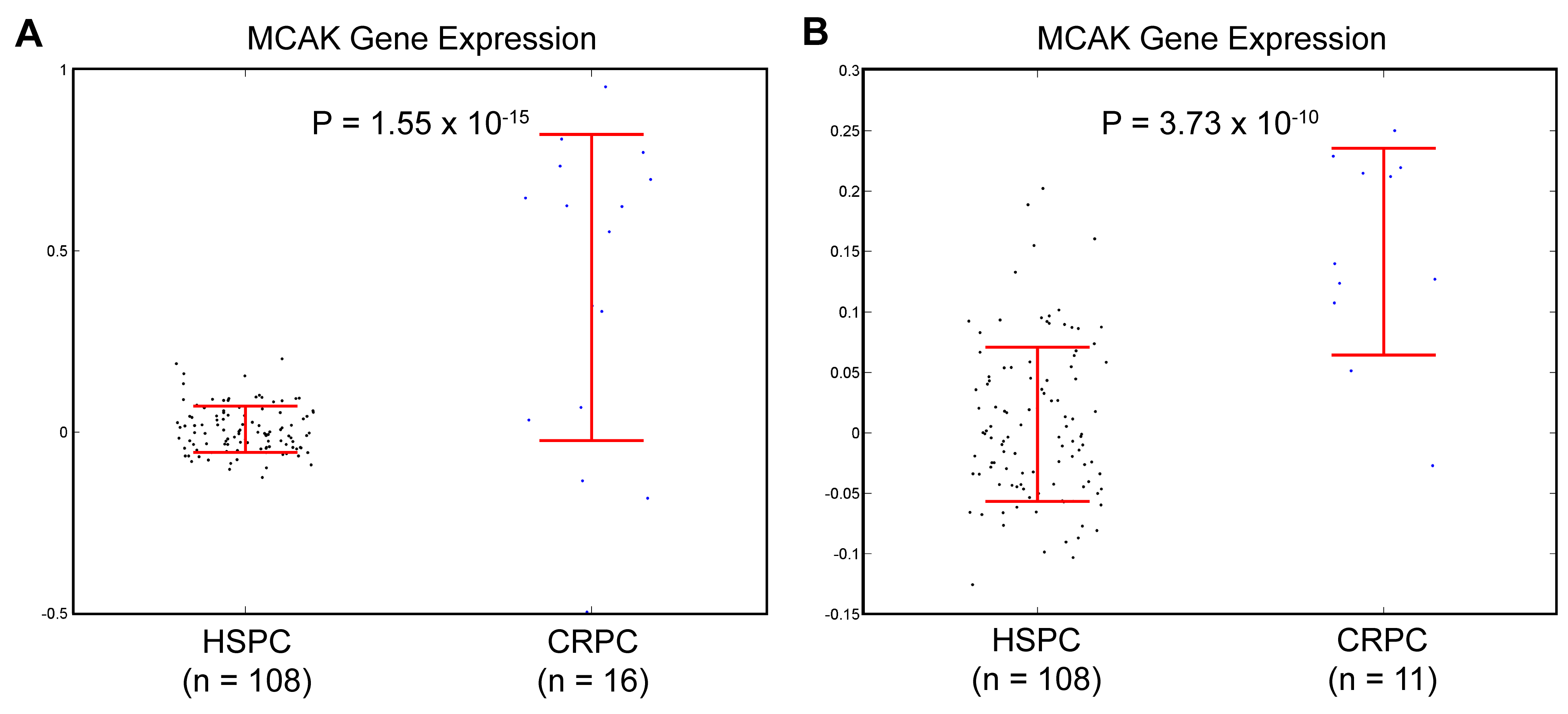

Supplement: Figure S2 — Increased MCAK transcript levels with progression to CRPC. (A) MCAK shows transcriptomic upregulation in localized CRPC (P = 1.55×10^−15^) and (B) metastatic CRPC (P = 3.73×10^−10^) compared to HSPC. (TIF) [file pone.0031259.s002.tif]

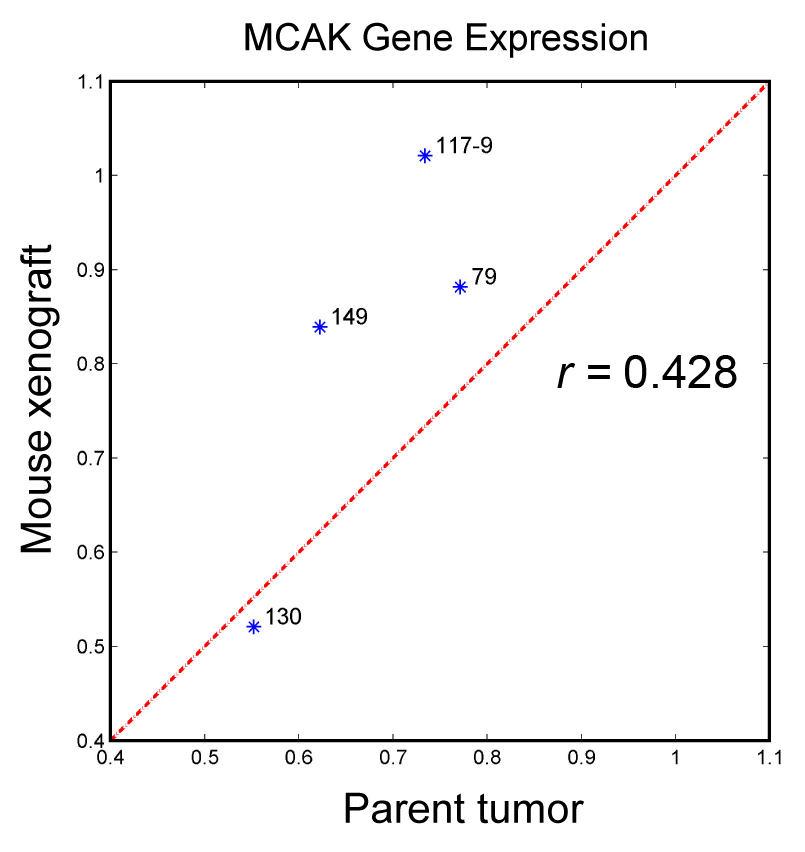

Supplement: Figure S3 — Concordance of MCAK gene expression between CRPC mouse xenografts (MDA-79, MDA-117, MDA-130, MDA-149) and their corresponding human parent tumors ( r = 0.428). (TIF) [file pone.0031259.s003.tif]

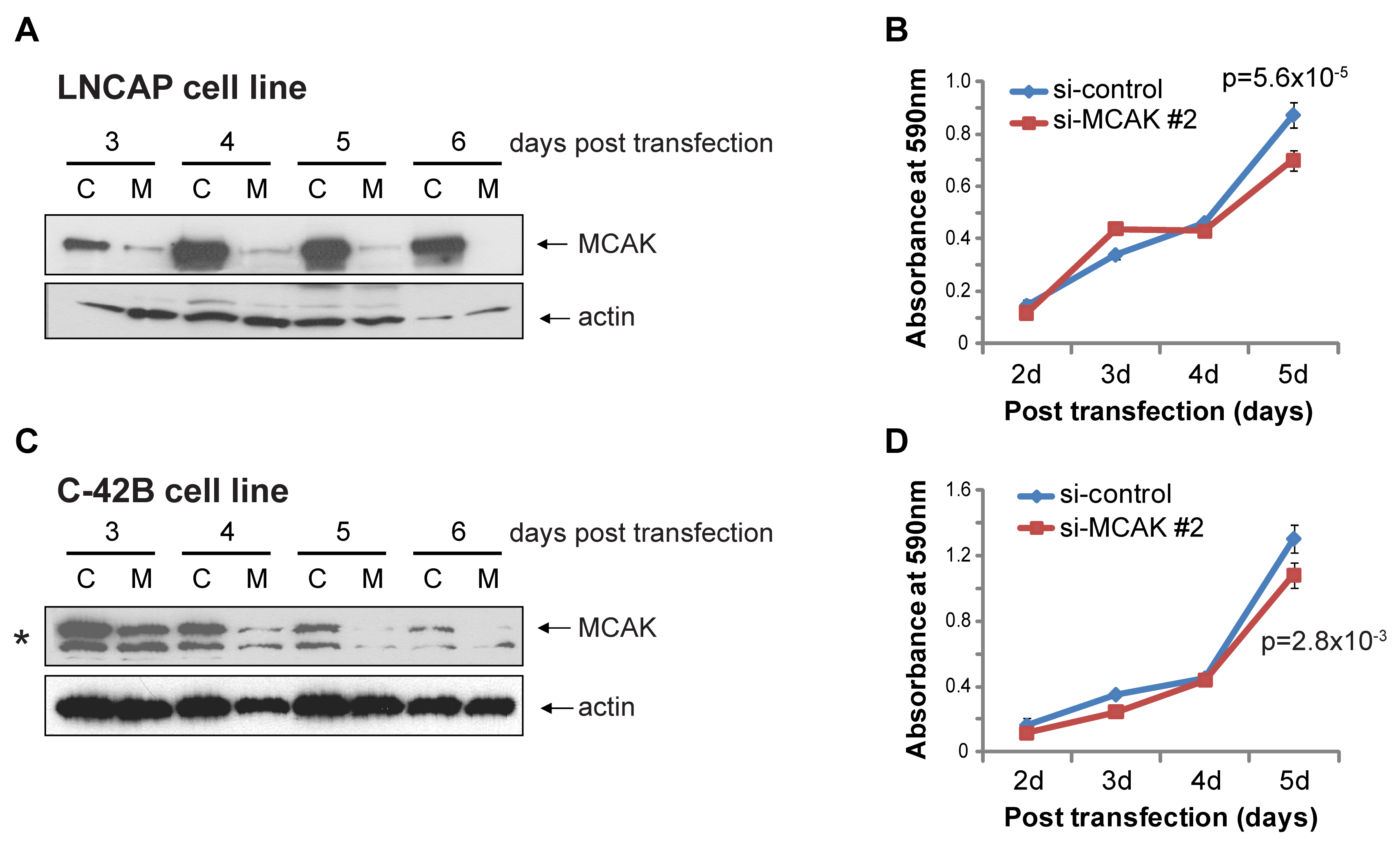

Supplement: Figure S4 — Growth inhibition of LNCaP and C4-2B cells by si-MCAK #2. A) Western blot confirming knockdown of MCAK by si-MCAK #2. Whole-cell lysates from LNCAP cells transfected with si-control (C) or si-MCAK #2 (M) were collected at different time points after transfection, as indicated. Actin was used as the loading control. B) MTT cell growth assays. LNCaP cells were treated the same as in A), and after a 24 h transfection, 4,000 cells were seeded into each well of a 96-well plate (n = 6 for each group) and followed by MTT assay. C) Western blot confirming knockdown of MCAK with si-MCAK #2. Whole-cell lysates from C4-2B cells transfected with si-control (C) or si-MCAK #2 (M) were collected at different time points after transfection, as indicated. Actin was used as the loading control. * indicates non-specific bands. D) MTT cell growth assays. C4-2B cells were transfected with si-control and si-MCAK #2, respectively. After a 24 h transfection, 4,000 cells were seeded into each well of a 96-well plate and incubated for different time periods, as indicated, followed by the MTT assay (n = 5 for each group). (TIF) [file pone.0031259.s004.tif]
