# Supplementary material for: Mitosis Phase Enrichment with Identification of Mitotic Centromere-Associated Kinesin As a Therapeutic Target in Castration-Resistant Prostate Cancer
Source: PLoS One. 2012 Feb 17;7(2):e31259. doi: 10.1371/journal.pone.0031259 (PMC3281954; doi:10.1371/journal.pone.0031259)
Supplement: Table S1 — Abbreviations: CXPX – cystoprostatectomy; PE – pelvic exenteration; TURP – transurethral resection; TAB – total androgen blockade (lupron + casodex); LHRH – lupron; ORCH – bilateral orchiectomy; XRT – radiation therapy. * Small cell carcinoma component was profiled from a mixed histology small cell/adenocarcinoma tumor. (DOC) [file pone.0031259.s005.doc]

**Table S1: Clinical data of molecularly profiled CRPC samples**

| **Patient** | **Histology** | **Procedure** | **PSA (CRPC)** | **ADT** | **XRT** | **Docetaxel** | **Other therapies** |
| --- | --- | --- | --- | --- | --- | --- | --- |
| 1 | ADENO-CA | CXPX | 176.4 | TAB | NO | YES | Estramustine, Immunotherapy |
| 2 | ADENO-CA | PE | 37.7 | TAB | YES | NO | Estramustine, Adriamycin, Vinblastine, Ketoconazole |
| 3 | ADENO-CA | CXPX | 8.5 | TAB | YES | YES | Carboplatin, Cyclophosphamide |
| 4 | ADENO-CA | CXPX | 4.7 | LHRH | YES | YES | Prednisone |
| 5 | Small Cell CA | AUTOPSY | 0.05 | TAB | NO | YES | Carboplatin, Cisplatin, Etoposide, Velcade |
| 6 | ADENO-CA | CXPX | 14.3 | TAB | NO | YES | Estramustine, Cyclophosphamide, Vincristine, Dexamethasone |
| 7 | ADENO-CA | CXPX | 6.3 | TAB | NO | YES |  |
| 8 | ADENO-CA | PE | 30.4 | LHRH | YES | YES | Kave |
| 9 | ADENO-CA | CXPX | 20.7 | TAB | NO | YES | Mitoxantrone, Prednisone, Kave |
| 10 | ADENO-CA | CXPX | 2 | LHRH | NO | YES |  |
| 11 | ADENO-CA | CXPX | 35.4 | ORCH | NO | NO | Kave |
| 12 | ADENO-CA | CXPX | 14.5 | ORCH | NO | YES | Estramustine |
| 13 | ADENO-CA | CXPX | 64.8 | TAB | NO | YES | Carboplatin, Dexamethasone, Thalidomide |
| 14 | Small Cell CA* | CXPX | 15.3 | LHRH | YES | NO |  |
| 15 | ADENO-CA | PE | 0.3 | TAB | YES | YES |  |
| 16 | Small Cell CA | PE | 0.05 | LHRH | YES | YES | Carboplatin, Etoposide |
| 17 | Small Cell CA | PE | n/a | ORCH | YES | YES |  |
| 18 | ADENO-CA | CXPX | 1.6 | LHRH | NO | YES | Cyclophosphamide, Vincristine, Dexamethasone |
| 19 | ADENO-CA | TURP | 920 | LHRH | NO | YES |  |
| 20 | ADENO-CA | CXPX | 3.9 | TAB | NO | YES | Carboplatin, Ketaconazole, Dexamethazone |

Abbreviations: CXPX – cystoprostatectomy; PE – pelvic exenteration; TURP – transurethral resection; TAB – total androgen blockade (lupron + casodex); LHRH – lupron; ORCH – bilateral orchiectomy; XRT – radiation therapy

* Small cell carcinoma component was profiled from a mixed histology small cell/ adenocarcinoma tumor.
