# Supplementary material for: Mitosis Phase Enrichment with Identification of Mitotic Centromere-Associated Kinesin As a Therapeutic Target in Castration-Resistant Prostate Cancer
Source: PLoS One. 2012 Feb 17;7(2):e31259. doi: 10.1371/journal.pone.0031259 (PMC3281954; doi:10.1371/journal.pone.0031259)
Supplement: Table S2 — Abbreviations: HSPC – hormone sensitive prostate cancer; HSPC-HG – hormone sensitive prostate cancer of high histologic grade (Gleason patterns 4/5); CRPC-adeno – castration resistant prostate cancer with adenocarcinoma histology; CRPC-SCC – castration resistant prostate cancer with small cell carcinoma histology. (DOC) [file pone.0031259.s006.doc]

**Table S2: Comparison of percent positive nuclear staining for phospho-histone H3 between different prostate cancer groups**

| **Group** | **Sample Size (# cases)** | **Sample Size (# cores)** | **Median**  **(Range)** | **Wilcoxon**  **rank-sum test**  **p-value** |
| --- | --- | --- | --- | --- |
|  |  |  |  |  |
| HSPC | 24 | 232 | 2.02% (0 – 100%) | <0.0001 |
| CRPC adeno | 41 | 325 | 4.04% (0 – 79.5%) |  |
|  |  |  |  |  |
| HSPC | 24 | 232 | 2.02% (0 – 100%) | <0.0001 |
| CRPC | 51 | 377 | 4.27% (0 – 100%) |  |
|  |  |  |  |  |
| HSPC-HG | 20 | 202 | 2.27% (0 – 100%) | <0.0001 |
| CRPC adeno | 41 | 325 | 4.04% (0 – 79.5%) |  |
|  |  |  |  |  |
| HSPC-HG | 20 | 202 | 2.27% (0 – 100%) | <0.0001 |
| CRPC SCC | 10 | 52 | 6.96% (0 – 100%) |  |
|  |  |  |  |  |
| HSPC-HG | 20 | 202 | 2.27% (0 – 100%) | <0.0001 |
| CRPC | 51 | 377 | 4.27% (0 – 100%) |  |
|  |  |  |  |  |
| CRPC adeno | 41 | 325 | 4.04% (0 – 79.5%) | 0.005 |
| CRPC SCC | 10 | 52 | 6.96% (0 – 100%) |  |
