# Supplementary material for: Mitosis Phase Enrichment with Identification of Mitotic Centromere-Associated Kinesin As a Therapeutic Target in Castration-Resistant Prostate Cancer
Source: PLoS One. 2012 Feb 17;7(2):e31259. doi: 10.1371/journal.pone.0031259 (PMC3281954; doi:10.1371/journal.pone.0031259)
Supplement: Table S3 — Abbreviations: HSPC – hormone sensitive prostate cancer; HSPC-HG – hormone sensitive prostate cancer of high histologic grade (Gleason patterns 4/5); CRPC-adeno – castration resistant prostate cancer with adenocarcinoma histology; CRPC-SCC – castration resistant prostate cancer with small cell carcinoma histology. (DOC) [file pone.0031259.s007.doc]

**Table S3: Comparison of percent positive cytoplasmic staining for gamma-tubulin between different prostate cancer groups**

| **Group** | **Sample Size (# cases)** | **Median**  **(Range)** | **Wilcoxon**  **rank-sum test**  **p-value** |
| --- | --- | --- | --- |
|  |  |  |  |
| HSPC | 56 | 5% (0% – 60%) | <0.0001 |
| CRPC-adeno | 41 | 25% (0% – 95%) |  |
|  |  |  |  |
| HSPC | 56 | 5% (0% – 60%) | <0.0001 |
| CRPC-SCC | 10 | 90% (5% –100%) |  |
|  |  |  |  |
| HSPC-HG | 23 | 5% (0% – 60%) | 0.008 |
| CRPC-adeno | 41 | 25% (0% – 95%) |  |
|  |  |  |  |
| HSPC-HG | 23 | 5% (0% – 60%) | 0.0003 |
| CRPC-SCC | 10 | 90% (5% – 100%) |  |
|  |  |  |  |
| CRPC-adeno | 41 | 25% (0% – 95%) | 0.002 |
| CRPC-SCC | 10 | 90% (5% – 100%) |  |
