# Supplementary material for: Mitosis Phase Enrichment with Identification of Mitotic Centromere-Associated Kinesin As a Therapeutic Target in Castration-Resistant Prostate Cancer
Source: PLoS One. 2012 Feb 17;7(2):e31259. doi: 10.1371/journal.pone.0031259 (PMC3281954; doi:10.1371/journal.pone.0031259)
Supplement: Table S4 — Abbreviations: HSPC – hormone sensitive prostate cancer; CRPC-adeno – castration resistant prostate cancer with adenocarcinoma histology; CRPC-SCC – castration resistant prostate cancer with small cell carcinoma histology. (DOC) [file pone.0031259.s008.doc]

**Table S4: Comparison of percent positive cytoplasmic staining for MCAK between different prostate cancer groups**

| **Group** | **Sample Size**  **(# cases)** | **Median**  **(Range)** | **Wilcoxon**  **rank-sum test**  **p-value** |
| --- | --- | --- | --- |
|  |  |  |  |
| HSPC | 38 | 52.5% (0% – 90%) | <0.0001 |
| CRPC-adeno | 41 | 75% (5%–100%) |  |
|  |  |  |  |
| HSPC | 38 | 52.5% (0% – 90%) | 0.007 |
| CRPC-SCC | 10 | 77.5% (5% – 100%) |  |
|  |  |  |  |
| HSPC | 38 | 52.5% (0% – 90%) | <0.0001 |
| CRPC | 51 | 75% (5% – 100%) |  |
|  |  |  |  |
| CRPC-SCC | 10 | 77.5% (5% – 100%) | 0.64 |
| CRPC-adeno | 41 | 75% (5% – 100%) |  |
|  |  |  |  |

Abbreviations: HSPC – hormone sensitive prostate cancer; HSPC-HG – hormone sensitive prostate cancer of high histologic grade (Gleason patterns 4/5); CRPC-adeno – castration resistant prostate cancer with adenocarcinoma histology; CRPC-SCC – castration resistant prostate cancer with small cell carcinoma histology.
